# Preclinical Drug Response Metric Based on Cellular Response Phenotype Provides Better Pharmacogenomic Variables with Phenotype Relevance

**DOI:** 10.3390/ph14121324

**Published:** 2021-12-17

**Authors:** Sanghyun Kim, Sohyun Hwang

**Affiliations:** 1 Department of Biomedical Science, College of Life Science, CHA University, Sungnam 13488, Korea; 2 Department of Pathology, CHA Bundang Medical Center, CHA University School of Medicine, Seongnam 13496, Korea

**Keywords:** high-throughput drug screening, drug response metrics, drug sensitivity metrics, dose-response assessment

## Abstract

High-throughput screening of drug response in cultured cell lines is essential for studying therapeutic mechanisms and identifying molecular variants associated with sensitivity to drugs. Assessment of drug response is typically performed by constructing a dose-response curve of viability and summarizing it to a representative, such as IC_50_. However, this is limited by its dependency on the assay duration and lack of reflections regarding actual cellular response phenotypes. To address these limitations, we consider how each response-phenotype contributes to the overall growth behavior and propose an alternative method of drug response screening that takes into account the cellular response phenotype. In conventional drug response screening methods, the ranking of sensitivity depends on either the metric used to construct the dose-response curve or the representative factor used to summarize the curve. This ambiguity in conventional assessment methods is due to the fact that assessment methods are not consistent with the underlying principles of population dynamics. Instead, the suggested phenotype metrics provide all phenotypic rates of change that shape overall growth behavior at a given dose and better response classification, including the phenotypic mechanism of overall growth inhibition. This alternative high-throughput drug-response screening would improve preclinical pharmacogenomic analysis and the understanding of a therapeutic mechanism of action.

## 1. Introduction

Drug responses in cultured cell lines are essential for identifying molecular features associated with therapeutic effectiveness of the drug, through integration with large genomic data [1,2,3,4,5,6]. This has been investigated by constructing a dose-response curve of a certain metric, such as viability, and summarizing the response curve into a representative quantity. The common summary factors are the concentration of half maximal inhibition (IC_50_) or half maximal effect (EC_50_) as a measure of potency, the maximal inhibition level (E_max_) as a measure of efficacy, and the area under the curve (AUC) as a measure of overall effectiveness. For better pharmacogenomic analysis, two limitations need to be addressed adequately: the issue of irreproducibility in drug response assays and the discordance between actual cellular response phenotypes and assessment metrics of drug response. Concerns regarding reproducibility in drug response studies are a practical issue raised repeatedly in preclinical studies [7,8,9]. On the other hand, the second limitation is a more general issue; while actual response phenotypes during drug treatment are diverse—including senescence and various forms of cell death—conventional metrics such as IC_50_ do not reflect such responses. Instead, these metrics are simply de facto standards for crudely categorizing cell lines as either sensitive or resistant without an underlying theoretical basis.

A recent multi-center study of the NIH Library of Network-Based Cellular Signatures Program (LINCS) on drug response assays [10] well evaluated the irreproducibility issue. A notable point of this study was that a growth rate [11,12]—instead of viability—was used as a metric for producing a dose-response curve. Viability always depends on the assay duration, and the duration is a relative factor for the doubling time of each cell line. To correct this confounder, this study used an alternative metric based on the apparent growth rate, under the assumption of a simple exponential cell growth [11,12]. Indeed, this metric reportedly improved the reproducibility and performance of pharmacogenomic association studies [13]. However, apparent growth-rate-based metrics still depend on the assay duration in numerous cases, as observed in the MCF10A cell line treated with neratinib in the LINCS study [10]. This implies that cellular growth behavior is not a simple exponential function.

Here, the widespread assumption for cellular growth, a simple exponential function, was reconsidered. We investigated how each cellular response phenotype upon a drug treatment contributes to the overall growth behavior and solved the equations of population dynamics of phenotypes. By using these population dynamics, we explored how the conventional method for drug response assessment itself—encompassing the assay duration, the metric for constructing a dose-response curve, and the summary factor—affects the assessment result of drug response and revealed its limitations for comparative analysis of drug response. In contrast, the alternative metric based on phenotype population dynamics produces time-independent characteristic quantities of drug response. This could provide better pharmacogenomic variables relevant to the response phenotype of the cell.

## 2. Results

### 2.1. Phenotype Dynamics Model: Each Cellular Response Phenotype Contributes Differently to the Overall Growth Behavior

**Figure 1:**
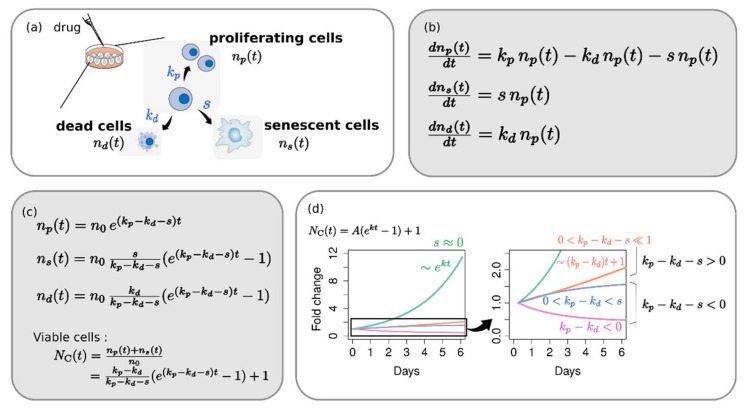
Phenotype dynamics model. (**a**) A diagrammatic illustration of phenotypic response of the cell upon a drug treatment. Proliferating cells can continue dividing, can enter a state of permanent cell cycle arrest, or can undergo cell death. (**b**) Differential equations for the population dynamics in each phenotype and (**c**) their analytical solutions. (**d**) Typical growth-curve shapes of viable cell depending on the phenotypic rate of change, $k_{p},k_{d},s$.

Phenotypes related to population growth can be divided into three groups: proliferating cells, dead cells, and cell cycle-arrested cells (i.e., senescent cells). Although senescence has never been considered individually in the conventional assessment of drug response, therapy-induced senescence is a widely reported phenomenon [14,15,16]. The number of cells in each phenotype ($n_{p}$ proliferating cells, $n_{s}$ senescent cells, and $n_{d}$ dead cells) changes due to cell division (with rate of change, $k_{p}$), cell death ($k_{d}$), or cell cycle arrest (s) of proliferating cells (Figure 1a). Accordingly, the complete set of differential equations for phenotype population dynamics is given, as shown in Figure 1b, and the explicit analytical solution (Figure 1c) is derived directly without numerical calculation. The death of senescent cells has been disregarded here unless there are senolytic drugs or immune cells.

The solution shows several notable points directly. All phenotypes have the same characteristic exponent in their growth function: $k=k_{p}-k_{d}-s$, which is the growth rate of proliferating cells. We call this just “growth rate (GR)” as distinct from “apparent growth rate (aGR)” that assumes a simple exponential growth of the total viable cells. Viable cell, $n_{p}+n_{s}$, does not follow a simple exponential form. Instead, it follows the function of time, $A\left(e^{kt}-1\right)+1$. Depending on $k_{p}$, $k_{d}$, and s, the shape of a growth curve changes from an increasing convex type ($k_{p}-k_{d}>s>0$) to linear ($k_{p}-k_{d}-s\ll 1$) or even concave ($s>k_{p}-k_{d}>0$) and to a decreasing convex type ($k_{p}-k_{d}<0$) (Figure 1d). However, it can be a simple exponential curve if *s*~0—that is, for the case of negligible senescence.

### 2.2. The Same Growth Curve Produces Different Dose-Response Curves Depending on the Assay Duration and the Metric

**Figure 2:**
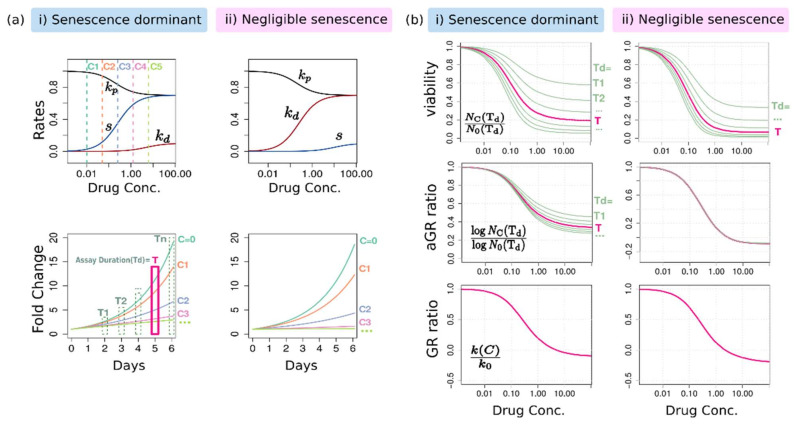
The dose-response curves of two distinct phenotype responses: (**i**) senescence-dominant and (**ii**) negligible-senescence. (**a**) The rates of change and the corresponding growth curves. The normal growth rate $k_{0}$ was assumed as 0.5 (that is, a doubling time = 1.4 days). (**b**) The dose-response curves of viability, aGR ratio, and also GR ratio.

By using the population dynamics of viable cells, we explored how the conventional dose-response curve changes depending on the assay duration and the metric used in producing a dose-response curve. To generate growth curves for certain drug–cell line pairs, we assigned the phenotypic rate of change for two cases depending on how dominantly the senescence occurs: (i) senescence-dominant or (ii) senescence-negligible. The phenotype rates of change as a function of dose and the growth curves at several doses are presented in Figure 2a. By using these same growth curves, we constructed dose-response curves of viability, aGR, and also GR at different end timepoints of the assay (Figure 2b). If dose-response curves are overlapped together, it means that the used metric for such dose-response curves does not depend on the assay duration.

Figure 2b shows clearly that different assay durations produce different dose-response curves. For the viability-based dose-response, this time-dependency is evident regardless of how significantly senescence occurs (Figure 2b upper). Both potency and efficacy are changed systematically; the longer duration gives the more sensitive response. However, the aGR-based curve is time-independent when senescence is negligible (Figure 2b middle). Otherwise, this also depends on the assay duration, but its variation is less than that of viability-based curve. As a matter of course, the GR (that is, $k_{p}-k_{d}-s$)-based dose-response curve is independent of the assay duration (Figure 2b lower).

### 2.3. Assay-Duration-Dependency of Drug Response Causes Significant Uncertainties in Summary Factors

**Figure 3:**
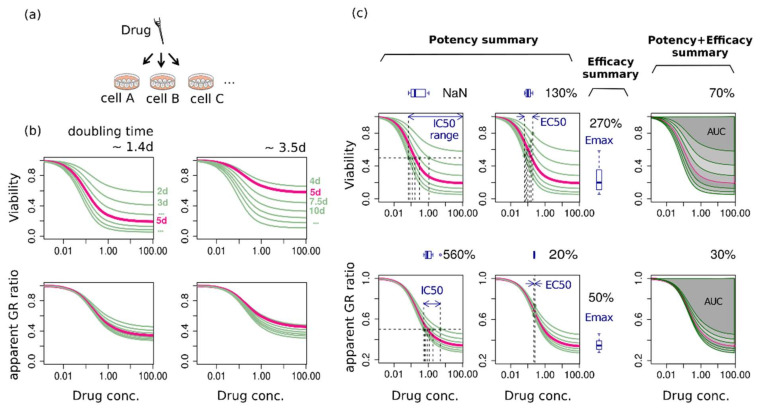
Time-dependency of drug response. (**a**) A diagrammatic illustration of the response classification assay of various cell lines upon a certain drug. (**b**) Drug responses of two cell lines that have the same GR ratio but different doubling times. (**c**) Variation of summary factors (IC_50_, EC_50_, E_max_, AUC) along the assay duration. The box plot, along with the ratio of the maximum deviation to the mean, shows uncertainty of summary factors by time-dependency.

Next, we considered what this time-dependency of the dose-response curve implies in a comparative study of drug response, e.g., a situation of categorizing cell lines to either be sensitive or resistant upon a certain drug. The notable point regarding the assay duration dependency is that each cell line is differently influenced by even the same duration of treatment depending on its own doubling time. This can be clearly shown in comparing dose-response curves of two example cell lines that show the same response to a certain drug in terms of cell cycle (Figure 3b). It was assumed that two cell lines had the same GR ratio (that of senescence-dominant case in Figure 2a(i) but with different doubling times (either ~1.4 days or ~3.5 days). The dose-response curves constructed in the same assay duration show that the cell line having the shorter intrinsic doubling time produced the more sensitive response curve (Figure 3b). Therefore, dose-response curves of each cell line are relatively biased, thereby implicating that there exists an intrinsic uncertainty in drug responses. In summary, variability in the doubling time of cell lines and a time-dependency of the dose-response make a comparative multi-assay difficult to be controlled consistently in terms of assay duration.

To estimate uncertainty of the summary factors due to time-dependency in a typical experimental condition, we summarized dose-response curves of various assay duration into IC_50_, EC_50_, E_max_, and AUC (Figure 3c). The duration ranged from 2 to 8 days, correspond to 1.5~6 times of the original doubling time, 1.4 days, of the cell having $k_{0}$ = 0.5. Then, the relative uncertainty was calculated as the ratio of the maximum deviation to the mean. As expected, viability-based metrics exhibit larger uncertainty than an aGR-based one, again (Figure 3c). Among the summary factors, IC_50_ is largely deviated along the assay duration and even to infinity in a 2-day assay. The AUC shows a relatively small deviation in both viability- and aGR dose-response curve.

### 2.4. Effectiveness Ranking Based on a Dose-Response Curve Depends on the Assessment Method

**Figure 4:**
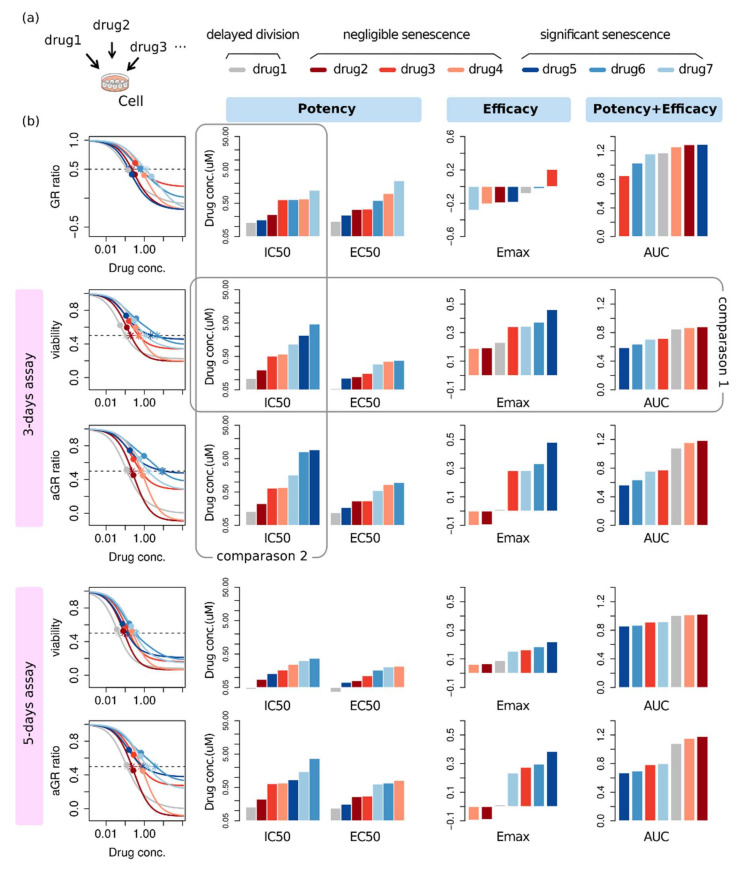
Ambiguity in assessing therapeutic effectiveness by conventional metrics. (**a**) A diagrammatic illustration of the assay. (**b**) For each dose-response curve, the summary factors, IC_50_, EC_50_, E_max_, and AUC were extracted. The stars and the circles on dose-response curves correspond to IC_50_ and EC_50_, respectively.

We considered the second situation of drug screening: assessing therapeutic effectiveness of various drugs for a certain cell line. In this multi-assay, there is no variability of intrinsic doubling time. The question would be either which drugs are effective and which are not, or how to determine the most effective drug to a given cell line? The common way to answer this question is to sort the summary factor. To this end, we investigated the therapeutic effectiveness of seven example drugs with various therapeutic effects in terms of dominant phenotype and potency and efficacy. For each drug, several growth curves were generated at several dosage, and these growth curves produced different dose-response curves depending on the metric and the assay duration. Summary factors were extracted from each dose-response curve and displayed in bar plots alongside the corresponding dose-response curve (Figure 4). In a conventional screening procedure, lower values of IC_50_, EC_50_, and E_max_ correspond to the more sensitive response, whereas higher values for AUC are more sensitive.

Overall, the assessment result for therapeutic effectiveness shows considerable ambiguity. For example, different summary factors produce different orders for therapeutic effectiveness: According to IC_50_ and EC_50_, the most effective drug is drug 1 (grey), while it is drug 4 (apricot) by E_max_, and drug 2 (dark red) by AUC based on a 3-day viability dose-response curve (Figure 4, comparison 1). Even with the same summary factor, an effectiveness ranking of a drug is different depending on which metric is used for producing a dose-response curve: When summarized into IC_50_, the 3-day viability dose-response curve gives drug 6 (blue) as the least effective one among seven drugs, while aGR gives the same drug as 6th, and GR gives it as 5th (Figure 4, comparison 2). Moreover, even with the same metric and the same summary factor, again a different assay duration produces a different order for therapeutic effectiveness (Figure 4, 3-day vs. 5-day).

### 2.5. Alternative Phenotype Metric Provides Time-Independent Characteristic Quantities of Drug Response

The above results indicate that the conventional drug response metrics have significant limitations for comparative analysis of drug response. Therefore, here we propose to measure phenotypic rate of change at a certain dose rather than constructing a full-range dose-response curve. Dead cell count to the total increased number of cells $\left(\frac{N_{d}}{N_{d-}N_{0+}N_{s}+N_{d}}=\frac{k_{d}}{k_{p}}\right)$, and senescence cell count to total increased number of viable cells $\left(\frac{N_{s}}{N_{p}-N_{0}+N_{s}}=\frac{s}{k_{p}-k_{d}}\right)$, along with GR, provide the system of linear equations that would enable the determination of all three unknowns: $k_{p}$, $k_{d}$, s.

These findings were confirmed by evaluating real data. We used a publicly available dataset that included both time-lapse enumerations of viable cells and end-point measurements of the phenotype fractions; that is, the ovarian cancer cell lines OV1369(R2) and OV1946 were treated with the PARP inhibitor olaparib for 6 days [17]. First of all, dose-response curves of viability and aGR displayed significant differences between 3-day and 6-day assays, indicating their time-dependency (Figure S1 and Supplementary Table S1a,b). The IC_50_ of the 6-day assays were much lower than those of 3-day assays (1.5–3 times for OV1369(R2) and ~10 times for OV1946).

**Figure 5:**
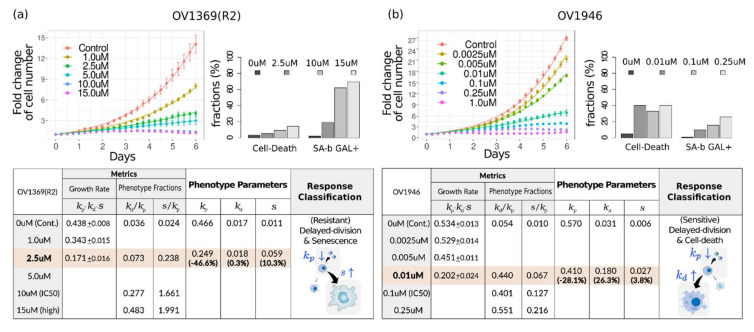
Evaluation of phenotype metric for ovarian cancer cell lines (**a**) OV1369(R2) and (**b**) OV1946 treated with olaparib. Fold change of viable cells and the fraction of dead and senescent cells were re-plotted using public raw data (upper). Calculation of the phenotype parameters and classification of drug response are summarized in the table (lower).

For the phenotype metric, GR was obtained by nonlinear fitting of the viable cell count. For the growth curves of the lowest 3~4 doses, the fitting converged well. The fraction of phenotype increase was obtained by reformulating the conventional phenotype fraction measured by β-Gal staining or flow cytometry (Figure 5). In the original paper [17], the phenotype fractions were measured just at four doses. Therefore, the phenotype parameters could be determined at a single concentration point where both GR and phenotype fractions were determined, along with at the drugless condition (Tables in Figure 5). At that concentration, 2.5 μM for OV1369(R2) and 0.01 μM for OV1946, olaparib showed cytostatic effects to each cell line. If we would focus on the response at 1 μM, OV1369(R2) is resistant because $k/k_{0}$ is larger than 0.5 at that concentration. In contrast, OV1946 is sensitive to olaparib because $k/k_{0}$ = 0.202/0.534 is smaller than 0.5 even at a much lower dose (0.01 μM). The dominant mechanism underlying growth inhibition can be classified according to the variation extent of each phenotypic rate of change relative to the normal growth rate. In the response of OV1396(R2) upon olaparib treatment, senescence is dominant (10.3% for senescence >0.3% for cell death) in addition to delayed cell division. Regarding OV1946 cells, the alternative phenotypic metric shows that even at a low dose of olaparib, growth is significantly inhibited, as well as this effect being due to cell death rather than senescence (26.3% for cell death >3.8% for senescence).

## 3. Discussion

We explored two different types of drug response metric under the consideration of phenotypic population dynamics: the conventional one based on a dose-response curve and the alternative phenotype metric evaluated at a single dose.

The drug response has been typically represented by summary factors extracted from a dose-response curve. The most widely used metric is viability. However, the difference in cell counts between drug-treated and drug-untreated conditions increases with time; that is, a longer treatment results in lower cell viability. This tendency becomes more prominent with an increase in dosage; hence, the longer the assay duration, the more sensitive the dose-response curve. Moreover, the assay duration is a relative factor for the intrinsic doubling time of each cell line, which means that a viability-based metric has a serious limitation for comparing the drug responses of various cell lines. In this regard, the growth rate could be a reasonable alternative to viability [11,12].

However, if the growth rate is derived on the assumption of a simple exponential growth even under drug treatment, the dose-response curve might still depend on time. A simple exponential growth results from the premise that the rate of cell number changes is proportional to the number of cells in each given time point. The proportional factor is the growth rate. If some of the viable cells are undergoing cell cycle arrest—which does not contribute to changes in the cell count—viable cells do not follow simple exponential growth. The number of proliferating cells itself might be an exponential function; however, the total number of viable cells (including both proliferating cells and cell cycle-arrested cells) is not. This indicates the need for considering the actual cellular response phenotypes when evaluating drug responses.

In this regard, we considered how each cellular response phenotype contributes to the population dynamics. Indeed, the viable cell count was not a simple exponential function but $A\left(e^{kt}-1\right)+1$. It indicates that time-dependency of the aGR is intrinsic because of the senescent cell subpopulations, regardless of other possible confounders mentioned in the literature [10]. On the contrary, GR ($k=k_{p}-k_{d}-s$) is time-independent, and it is the only characteristic exponent in the population dynamics. Unless specifically enumerating the proliferating cells, a proper fitting model, not a simple exponential function, should be applied to the number of either viable cells ($\frac{N_{p}+N_{s}}{N_{0}}=A\left(e^{kt}-1\right)+1$) or dead cells ($\frac{N_{d}}{N_{0}}=B\left(e^{kt}-1\right)$ to determine GR. In a therapeutically effective dose where the growth is too small, measuring dead cell counts would be better for the convergence of nonlinear fitting. Recent advancements in time-lapse imaging in live cell microscopy [18] potentially provide a useful platform for measuring the cellular kinetics to determine the growth rate.

The first obvious limitation of the conventional drug response metrics for response classification of cell lines arises from the assay-duration dependency of the dose-response curve and doubling-time variability of cell lines. This is an additional uncertainty to other experimental variabilities such as seeding numbers, effects from either a direct cell counting or CellTiter-Glo assay, and edge effects in a well plate [10,11]. Extracting response summaries from two types of dose-response curves along the assay duration exhibits that summary factors from an aGR-based dose-response curve have a smaller uncertainty than those from a viability-based curve. Among the widely used summary factors, EC_50_ has the smallest deviation, but it does not represent an overall effectiveness, only potency. Efficacy is captured separately as E_max_. Instead, AUC captures an overall effectiveness as well as showing a relatively small deviation. The most common factor, IC_50_, is the worst in terms of uncertainty, as was also shown in a statistical framework study that used uncertainty estimations to improve biomarker discovery with assessment of cell line drug response [19].

However, even in the case of drug screening for a single cell line wherein there is no doubling time variability, the dose-response curve-based assessment has intrinsic ambiguity. This is the order of the therapeutic effectiveness changes depending on the metric (viability or aGR or even GR) for a dose-response curve and the summary factor, and the length of the assay, in case of the conventional metric. The ambiguity of therapeutic effectiveness assessment using a dose-response curve is represented even in the GR dose-response curve—that is, being constructed with a time-independent, characteristic growth rate. This is intrinsic in that summarizing a dose-response curve is customarily accomplished by fitting with an empirical sigmoidal curve. In the absence of a theoretical basis, the conventional summary factors are merely apparent quantities, similar to how simple exponential fitting provides apparent growth rates for viable cell growth rather than a characteristic exponent. Furthermore, due to uncertainty propagation, summarizing into apparent quantities may result in greater ambiguity than the original uncertainty by experimental variability [19]. This might be a reason for not only dimness and obscurity within a pharmacogenomic study but also inconsistency or a modest correlation between large pharmacogenomic studies such as CCLE [2], GDSC [20], and CTRP [21]. This finding is in line with a previous report regarding systematic variations of the summary factors in large-scale drug response data [22]. In that study, different summary factors captured distinct information, and the most informative factor varied with the drug. Therefore, this study concluded that factors other than potency should be considered in the comparative analysis of drug response, particularly at clinically relevant concentrations. However, while the general cellular growth as a function of time is a derivative form of exponential function, which has a characteristic exponent, how about this characteristic quantity itself as a function of dose? Unlike in the case of a growth curve, it is difficult to expect that the dose-response curve has an analytical functional form or characteristic quantity in general. Classical pharmacology is not ready to explain a dose-response with a theoretical basis.

In this regard, we suggest an alternative evaluation of drug response, that is, one not based on a dose-response curve but accounts for the response at a single dose as it is. GR alone is not enough for an assessment of drug response because there are various combinations of $k_{p},\;k_{d},\;s$ for the same GR. We want a response variable and classification to have phenotypic relevance. We need to measure all phenotype rates that contain all the information on the drug response at a given dose and are directly connected to the mechanism underlying growth inhibition.

**Figure 6:**
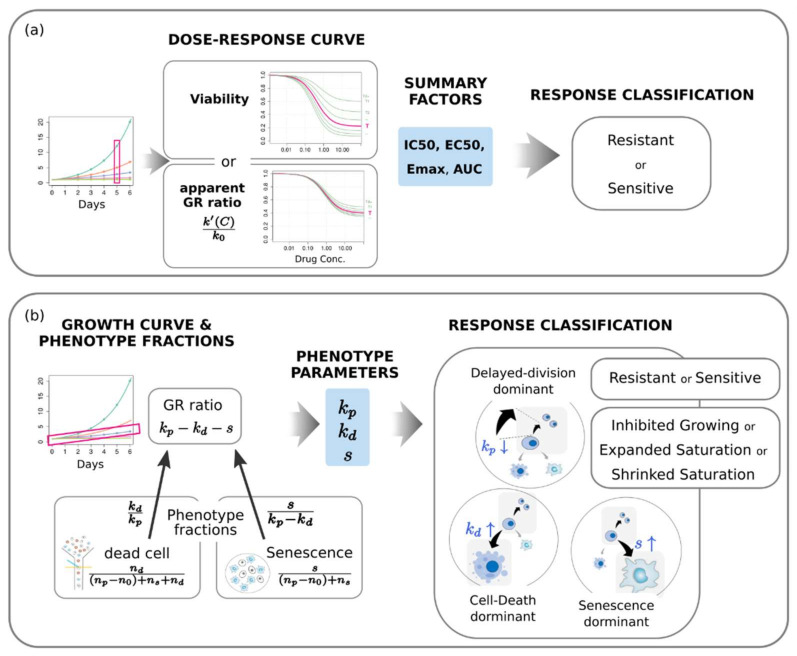
Evaluation of drug response based on (**a**) the conventional dose-response curve and (**b**) the phenotype population dynamics at a single dose. Three types of response classification are possible with the phenotype metric.

This phenotype metric provides clear phenotype-relevant pharmacogenomic variables. The therapeutic effect is categorized either as cytotoxic or cytostatic, and the dominant phenotype either as delayed division, senescence, or death. Only in the case of $k_{p}-k_{d}<0$ is the effect cytotoxic (that is, actual decrease in viable cells); otherwise, it is cytostatic (inhibited growth). Even if $k_{p}-k_{d}-s$ is negative, in the case of $s>k_{p}-k_{d}>0$, the viable cells increase until they all finally become senescent cells. Then, the fold change of the viable cells reaches to a certain saturation level $\frac{s}{s-\left(k_{p}-k_{d}\right)}>1$. Therefore, the detailed classification is either inhibited growing ($k_{p}-k_{d}>s>0$), expanded saturation ($s>k_{p}-k_{d}>0$), or shrunk saturation ($k_{p}-k_{d}<0$). Note that unless s≈0, a complete shrinkage does not happen because the fold change of viable cells becomes a nonzero level $\frac{s}{s-\left(k_{p}-k_{d}\right)}<1$. Classification of the dominant phenotype can be performed by comparing $\frac{s}{k_{0}}$, $\frac{k_{d}}{k_{0}}$, and $\frac{k_{0}-k_{p}}{k_{0}}$. The classical categorization into either sensitive or resistant can be applicable in line with a de facto classification by IC_50_—that is, classifying by whether or not the GR ratio ($k/k_{0}$) is larger than 0.5 at a clinically relevant dose. Evaluation flows for the conventional metrics and the alternative phenotype metric are summarized in Figure 6.

The significance of senescence in drug response is notable. Growth behavior that deviates from simple exponential functions and the absence of complete shrinkage of viable cells are certainly the consequence of senescence. Furthermore, senescence causes systematic variation in a conventional dose-response curve; as the senescence becomes more pronounced, the dose-response curve appears less sensitive even for the same proliferating growth rate. This is because senescence reduces the proliferating growth rate, but it does not reduce viability. If an assay duration increases, then the tendency for a decreased sensitivity along senescence is mitigated.

We believe the ability of the phenotype metric to provide the characteristic quantities of drug response and the mechanism underlying growth inhibition would markedly improve pharmacogenomic analysis. This improvement of the pharmacogenomic variables in preclinical pharmacology will provide better translation into clinical studies and useful information for treatment decision-making. Another advantage of this phenotypic metric is that it does not require analysis in a wide range of dose. In addition to a drugless control assay, a single-concentration assay at a clinically relevant concentration is sufficient. Of course, evaluations in a wider range of concentrations might give a separate dose-response curve for each of $k_{p},\;k_{d},\;s$, which would provide rich information regarding therapeutic mechanisms of action.

Conventional dose-response curves have some limitations for providing precise pharmacogenomic variables, but their usefulness is undeniable for qualitatively visualizing the overall drug response of a single cell-line upon a certain molecular feature. For example, ML239 cytotoxicity in NCI H661 LCLC cells along the knockdown efficiency of *FADS2* were well visualized in the viability dose-response curve [6]. Therefore, we conclude that it is appropriate to use the phenotype metric for therapeutic effectiveness assessment related to pharmacogenomic association studies and the dose-response curve for qualitative visualization of overall drug response.

## 4. Methods

### 4.1. Exploring the Conventional Evaluation Method of Drug Response

Phenotype Parameters and Growth Curve: Growth curves for a certain drug–cell line pair were generated by assigning phenotypic rate of change in the population dynamics model. The rate of change was assumed respectively as Hill function of dose, which is the most widely used functional form for dose response and even for a growth rate [11].

$$k_{p}=k_{0}\left(1-\frac{k_{mp}C}{HC_{50p}+C}\right)\;k_{d}=k_{0}\frac{k_{md}C}{HC_{50d}+C}\;s=k_{0}\frac{s_{m}C}{HC_{50s}+C}$$

Here, the subscript *m* means a maximum effect for each parameter; e.g., $k_{md}$ is the maximum effect for $k_{d}$, relative to the normal growth rate, $k_{0}$. The growth rate under drug-less condition ($k_{0}$) and the intrinsic doubling time ($T_{d}$) are related by the relation, $k_{0}\cdot T_{d}=ln\;2$. HC_50_ is the concentration corresponding to the half maximal effect for each parameter. These parameter sets determine the growth behavior of each phenotype completely according to the phenotype population dynamics (Figure 1b,c). The growth curve is presented as a fold change ($N_{C}$) of viable cells—that is, a sum of proliferating cell ($n_{p}$) and senescent cell ($n_{s}$) at a certain dose *C*. $N_{0}$ is the fold change in normal condition, i.e., a drug-less condition (*C* = 0).

Does–response Curve: For given growth curves for various doses, viability $=\frac{N_{C}\left(T\right)}{N_{0}\left(T\right)}$ and apparent growth rate $aGR=\frac{ln\;N_{C}\left(T\right)}{ln\;N_{0}\left(T\right)}$ were calculated at a certain time point (*T*), and these calculations generated a dose-response curve. Here, time difference between the drug injection and the time point at which viability or aGR is calculated, corresponds to the assay duration. Several dose-response curves were generated along the assay duration.

Response Summary: For a given dose-response curve of either viability or apparent growth rate, potency (IC_50_ and EC_50_) and efficacy (E_max_) were obtained by nonlinear fitting with a classical Hill function, $f\left(x\right)=c+\frac{d-c}{1+{\left(\frac{x}{e}\right)}^{b}}$. This was performed with the R package ”drm”, and it produced well-converged results except in high doses, where the growth was too small. The fitting parameter b and d were 1 in all cases. The summary factors were determined through

$$EC_{50}=e\;E_{max}=c\;IC_{50}=e\times {\left(\frac{d-0.5}{0.5-c}\right)}^{1/b}≅e\times \left(\frac{0.5}{0.5-c}\right)$$

The overall effectiveness was measured as the area under the inhibitory curve (=1-viability), AUC, by using the “trapz” function in R package “pracma”.

### 4.2. Drug Response Assessment of Public Data

Public Data: Drug response data were downloaded from the source data link (https://www.nature.com/articles/s41467-019-10460-1#Sec32) of Fleury’s paper [17] (download on 9 June 2020 accessed on 1 November 2021). This was the only publicly available dataset to date that included both time-lapse enumerations of viable cells and the end-point measurements of the phenotype fractions. We assessed drug responses of OV1369(R2) and OV1946 cell lines treated with olaparib through both of the metrics: the conventional metric and the alternative phenotype metric. All data analyses and plots were performed in R.

Growth Rates and Viability from Time-Lapse Cell Growth Measurements: The viable cell counts, divided by the initial number $n_{0}$, were applied to a 2-parameter exponential function, $A\left(e^{kt}-1\right)+1$. The nonlinear least squares regression was performed with the “nls” function in R. The proliferating growth rate, k, is given as one of the fitting parameters. For the conventional assessment, apparent growth rate was determined by either nonlinear fitting with a simple exponential function or by calculating aGR = $\frac{ln\;N_{C}\left(T\right)}{ln\;N_{0}\left(T\right)}$ for the assay duration of 3 days and 6 days. Viability $=\frac{N_{C}\left(T\right)}{N_{0}\left(T\right)}$ was measured for the same assay duration.

Phenotype Fraction: According to the explicit functional form of each phenotypic growth, the fractions of phenotype increase are given as $\frac{N_{s}}{N_{p}-N_{0}+N_{s}}=\frac{s}{k_{p}-k_{d}}$ for senescent cells and $\frac{N_{d}}{N_{d-}N_{0+}N_{s}+N_{d}}=\frac{k_{d}}{k_{p}}$ for dead cells. Note that $N_{p}-N_{0}$, instead of $N_{p}$, makes the fractions a simple ratio of the parameters. However, the phenotype fractions in the original data are the conventional forms measured by either senescence-associated β-galactosidase assay ($\frac{N_{s}}{N_{p}+N_{s}}$ for senescent cells) or flow cytometry ($\frac{N_{d}}{N_{p}+N_{s}+N_{d}}$ for dead cells). To convert the conventional phenotype fraction to the fraction of phenotype increase, a fold change (FC) of viable cells was used: $\frac{N_{s}}{N_{p}-N_{0}+N_{s}}=\frac{N_{s}}{N_{p}+N_{s}}\frac{FC}{FC-1}$ and $\frac{N_{d}}{N_{p}-N_{0}+N_{s}+N_{d}}=\frac{\frac{N_{d}}{N_{p}+N_{s}+N_{d}}FC}{FC-1+\frac{N_{d}}{N_{p}+N_{s}+N_{d}}}$ for FC= $\frac{N_{p}+N_{s}}{N_{0}}$ obtained from the kinetic measurements.

Phenotype Parameter and Response Classification: $k_{p}$, $k_{d}$, and s were determined by the system of linear equations; proliferating growth rate $k=k_{p}-k_{d}-s$ and the fractions of phenotype increase $\frac{N_{d}}{N_{d-}N_{0+}N_{s}+N_{d}}=\frac{k_{d}}{k_{p}}$ and $\frac{N_{s}}{N_{p}-N_{0}+N_{s}}=\frac{s}{k_{p}-k_{d}}$. Thereafter, at a certain drug concentration *C*, the variation extent of each parameter relative to $k_{p}\left(0\right)\approx k_{0}$ was calculated. For example, the relative change in $k_{d}$ is $\frac{k_{d}\left(C\right)-k_{d}\left(0\right)}{k_{0}}$. By comparing the relative change in each parameter, a dominant mechanism underlying growth inhibition could be determined.

## Data Availability

No new data were created in this study. The data that support the findings of this study are available in the source data file of Fleury’s work at https://www.nature.com/articles/s41467-019-10460-1#Sec32 (accessed on 1 November 2021).

## Supplementary Materials

The following are available online at https://www.mdpi.com/article/10.3390/ph14121324/s1, Figure S1: Time-dependence of the conventional metrics, Table S1: Detailed calculations in evaluation of the public data with each drug response metric.

## References

[1] Wang L., McLeod H.L., Weinshilboum R.M. (2011). Genomics and Drug Response. N. Engl. J. Med..

[2] Barretina J., Caponigro G., Stransky N., Venkatesan K., Margolin A.A., Kim S., Wilson C.J., Lehár J., Kryukov G.V., Sonkin D. (2012). The Cancer Cell Line Encyclopedia Enables Predictive Modelling of Anticancer Drug Sensitivity. Nature.

[3] Garnett M.J., Edelman E.J., Heidorn S.J., Greenman C.D., Dastur A., Lau K.W., Greninger P., Thompson I.R., Luo X., Soares J. (2012). Systematic Identification of Genomic Markers of Drug Sensitivity in Cancer Cells. Nature.

[4] Costello J.C., Heiser L.M., Georgii E., Gönen M., Menden M.P., Wang N.J., Bansal M., Ammad-Ud-Din M., Hintsanen P., Khan S.A. (2014). A Community Effort to Assess and Improve Drug Sensitivity Prediction Algorithms. Nat. Biotechnol..

[5] Iorio F., Knijnenburg T.A., Vis D.J., Bignell G.R., Menden M.P., Schubert M., Aben N., Gonçalves E., Barthorpe S., Lightfoot H. (2016). A Landscape of Pharmacogenomic Interactions in Cancer. Cell.

[6] Rees M.G., Seashore-Ludlow B., Cheah J.H., Adams D.J., Price E.V., Gill S., Javaid S., Coletti M.E., Jones V.L., Bodycombe N.E. (2016). Correlating Chemical Sensitivity and Basal Gene Expression Reveals Mechanism of Action. Nat. Chem. Biol..

[7] Haibe-Kains B., El-Hachem N., Birkbak N.J., Jin A.C., Beck A.H., Aerts H.J.W.L., Quackenbush J. (2013). Inconsistency in Large Pharmacogenomic Studies. Nature.

[8] Collins F.S., Tabak L.A. (2014). Policy: NIH Plans to Enhance Reproducibility. Nat. News.

[9] Freedman L.P., Cockburn I.M., Simcoe T.S. (2015). The Economics of Reproducibility in Preclinical Research. PLoS Biol..

[10] Niepel M., Hafner M., Mills C.E., Subramanian K., Williams E.H., Chung M., Gaudio B., Barrette A.M., Stern A.D., Hu B. (2019). A Multi-Center Study on the Reproducibility of Drug-Response Assays in Mammalian Cell Lines. Cell Syst..

[11] Hafner M., Niepel M., Chung M., Sorger P.K. (2016). Growth Rate Inhibition Metrics Correct for Confounders in Measuring Sensitivity to Cancer Drugs. Nat. Methods.

[12] Harris L.A., Frick P.L., Garbett S.P., Hardeman K.N., Paudel B.B., Lopez C.F., Quaranta V., Tyson D.R. (2016). An Unbiased Metric of Antiproliferative Drug Effect In Vitro. Nat. Methods.

[13] Hafner M., Niepel M., Sorger P.K. (2017). Alternative Drug Sensitivity Metrics Improve Preclinical Cancer Pharmacogenomics. Nat. Biotechnol..

[14] Ewald J.A., Desotelle J.A., Wilding G., Jarrard D.F. (2010). Therapy-Induced Senescence in Cancer. JNCI J. Natl. Cancer Inst..

[15] Mikuła-Pietrasik J., Niklas A., Uruski P., Tykarski A., Książek K. (2020). Mechanisms and Significance of Therapy-Induced and Spontaneous Senescence of Cancer Cells. Cell. Mol. Life Sci..

[16] Wang B., Kohli J., Demaria M. (2020). Senescent Cells in Cancer Therapy: Friends or Foes?. Trends Cancer.

[17] Fleury H., Malaquin N., Tu V., Gilbert S., Martinez A., Olivier M.-A., Sauriol A., Communal L., Leclerc-Desaulniers K., Carmona E. (2019). Exploiting Interconnected Synthetic Lethal Interactions between PARP Inhibition and Cancer Cell Reversible Senescence. Nat. Commun..

[18] Gelles J.D., Mohammed J.N., Santos L.C., Legarda D., Ting A.T., Chipuk J.E. (2019). Single-Cell and Population-Level Analyses Using Real-Time Kinetic Labeling Couples Proliferation and Cell Death Mechanisms. Dev. Cell.

[19] Wang D., Hensman J., Kutkaite G., Toh T.S., Galhoz A., Dry J.R., Saez-Rodriguez J., Garnett M.J., Menden M.P., GDSC Screening Team (2020). A Statistical Framework for Assessing Pharmacological Responses and Biomarkers Using Uncertainty Estimates. eLife.

[20] Yang W., Soares J., Greninger P., Edelman E.J., Lightfoot H., Forbes S., Bindal N., Beare D., Smith J.A., Thompson I.R. (2013). Genomics of Drug Sensitivity in Cancer (GDSC): A Resource for Therapeutic Biomarker Discovery in Cancer Cells. Nucleic Acids Res..

[21] Seashore-Ludlow B., Rees M.G., Cheah J.H., Cokol M., Price E.V., Coletti M.E., Jones V., Bodycombe N.E., Soule C.K., Gould J. (2015). Harnessing Connectivity in a Large-Scale Small-Molecule Sensitivity Dataset. Cancer Discov..

[22] Fallahi-Sichani M., Honarnejad S., Heiser L.M., Gray J.W., Sorger P.K. (2013). Metrics Other than Potency Reveal Systematic Variation in Responses to Cancer Drugs. Nat. Chem. Biol..

