# Supplementary material for: Preclinical Drug Response Metric Based on Cellular Response Phenotype Provides Better Pharmacogenomic Variables with Phenotype Relevance
Source: Pharmaceuticals (Basel). 2021 Dec 17;14(12):1324. doi: 10.3390/ph14121324 (PMC8707441; doi:10.3390/ph14121324)
Supplement: Supplementary file 1 [file pharmaceuticals-14-01324-s001.zip › pharmaceuticals-1472016-supplementary.pdf]

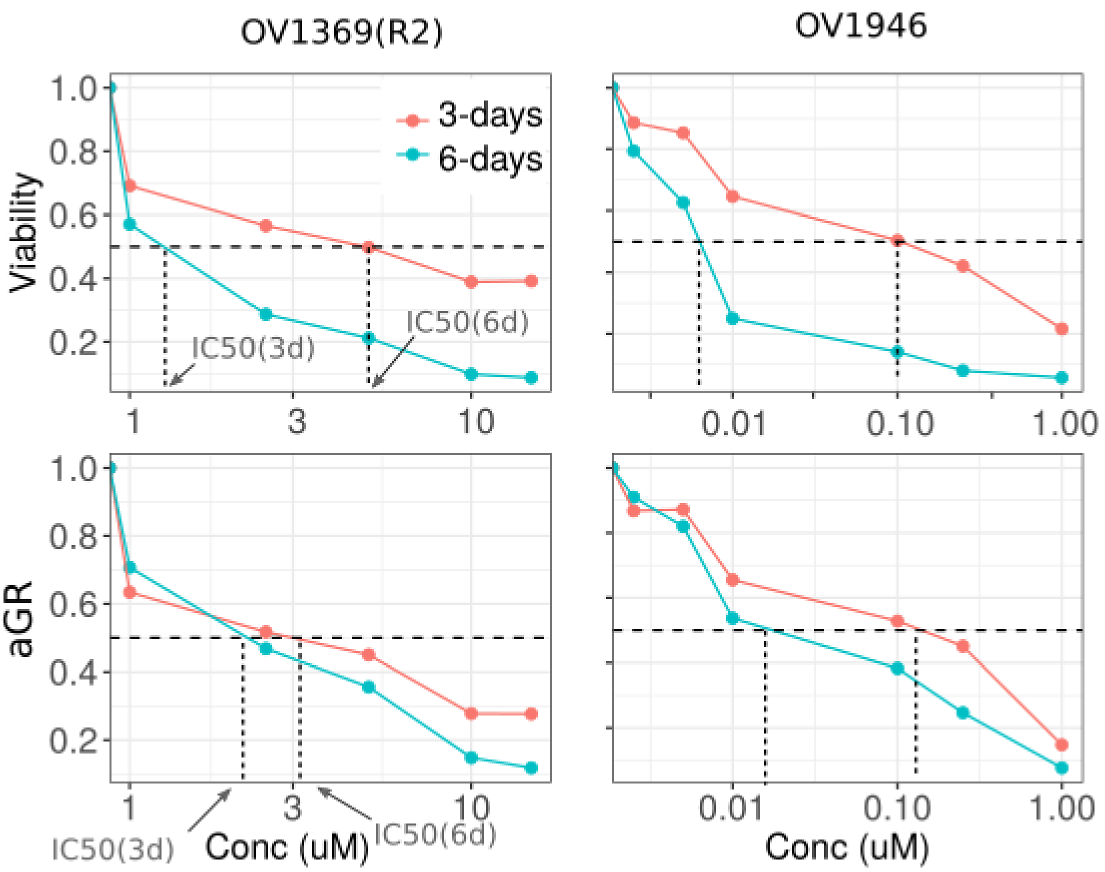

Figure S1. Time dependence of the conventional metrics.

Viability and aGR for different assay durations (3-days and 6-days) were compared for OV1369(R2) and OV1946 cells respectively. Detailed values of each metric evaluation are in Supplementary Table 1. Clearly 6-days assay displayed a lower viability in the most of concentration range, that is, a more sensitive response curve. For both viability- and aGR-dose-responses, the concentrations corresponding to the half maximal inhibition were lower in 6-days assay than in 3-days one.

Table S1. Detail calculations in evaluation of the public data with each drug-response metric.

a. Viability

Cell line	Conc. (uM)	Fold change at day 3	Fold change at day 6	Viability at day3	Viability at day6
OV1369(R2)	0.0	3.770	14.048	1.000	1.000
	1.0	2.605	8.021	0.691	0.571
	2.5	2.133	4.027	0.566	0.287
	5.0	1.878	2.982	0.498	0.212
	10.0	1.467	1.372	0.389	0.098
	15.0	1.476	1.220	0.392	0.087
OV1946	0.0000	5.234	27.554	1.000	1.000
	0.0025	4.638	21.890	0.886	0.794
	0.0050	4.463	17.250	0.853	0.626
	0.0100	3.382	6.894	0.646	0.250
	0.1000	2.638	3.890	0.504	0.141
	0.2500	2.204	2.200	0.421	0.080
	1.0000	1.132	1.566	0.216	0.057

b. Apparent growth rate (aGR) based metric

Cell line	Conc. (uM)	end point calculation		**simple exponential** fitting (**3 days** fitting)					**simple exponential** fitting (**6 days** full fitting)				
		aGR (day3)	aGR (day6)	apparent *k*(c)	std.error	t-value	p-value	**aGR (day3)**	apparent *k*(c)	std.error	t-value	p-value	**aGR (day6)**
OV1369(R2)	0.0	1.000	1.000	0.432	0.005	78.570	1.204E-17	1.000	0.445	0.001	487.717	1.792E-49	1.000
	1.0	0.649	0.727	0.306	0.005	60.314	2.853E-16	0.634	0.343	0.002	218.204	4.312E-41	0.707
	2.5	0.485	0.441	0.260	0.003	78.320	1.251E-17	0.517	0.247	0.002	118.704	9.422E-35	0.468
	5.0	0.390	0.332	0.232	0.007	34.266	2.429E-13	0.450	0.195	0.003	65.575	1.381E-28	0.356
	10.0	0.222	0.086	0.153	0.006	24.720	1.161E-11	0.278	0.088	0.007	13.487	1.074E-12	0.148
	15.0	0.226	0.054	0.152	0.007	22.572	3.382E-11	0.277	0.071	0.008	8.747	6.274E-09	0.118
OV1946	0.0000	1.000	1.000	0.541	0.006	96.676	1.003E-18	1.000	0.562	0.002	298.238	2.393E-44	1.000
	0.0025	0.901	0.906	0.488	0.009	56.285	6.524E-16	0.868	0.525	0.002	279.417	1.143E-43	0.910
	0.0050	0.871	0.813	0.489	0.006	78.848	1.154E-17	0.872	0.486	0.002	251.980	1.365E-42	0.820
	0.0100	0.666	0.497	0.393	0.006	67.419	7.527E-17	0.655	0.348	0.005	71.018	2.057E-29	0.537
	0.1000	0.501	0.328	0.331	0.005	64.301	1.327E-16	0.528	0.263	0.006	43.021	3.140E-24	0.383
	0.2500	0.392	0.179	0.290	0.008	37.772	7.622E-14	0.451	0.179	0.010	18.722	7.967E-16	0.247
	1.0000	0.053	0.098	0.107	0.020	5.266	1.990E-04	0.148	0.060	0.007	8.992	3.752E-09	0.077

c. Phenotype metric

Cell line	Conc. (uM)	Metric1. Overall growth rate (GR) by kinetic measurement				Metric2. Phenotype fractions		Resulting parameters			
		$k(c) = k_p - k_d - s$	std.error	t-value	p-value	k_d/k_p	s/k_p	k_p	kd	s	
OV1369(R2)	0.0	0.438	0.008	55.540	5.185E-26	0.036	0.024	0.466	0.017	0.011	
	1.0	0.343	0.015	23.575	1.304E-17						
	2.5	0.171	0.016	11.002	1.224E-10	0.073	0.238	0.249	0.018	0.059	
	5.0	0.029	0.014	2.072	4.971E-02						
	10.0					0.277	1.661				
	15.0					0.483	1.991				
								-46.625	0.251	10.341	% Change compared to k_0(c=0)
OV1946	0.0000	0.534	0.013	40.147	4.467E-22	0.054	0.010	0.570	0.031	0.006	
	0.0025	0.529	0.014	38.535	1.087E-21						
	0.0050	0.451	0.011	41.334	2.373E-22						
	0.0100	0.202	0.024	8.418	2.507E-08	0.440	0.067	0.410	0.180	0.027	
	0.1000	0.031	0.026	1.181	2.504E-01	0.401	0.127				
	0.2500					0.551	0.216				
	1.0000										
								-28.112	26.259	3.807	% Change compared to k_0(c=0)
